# Preclinical evaluation of [^18^F]SYN1 and [^18^F]SYN2, novel radiotracers for PET myocardial perfusion imaging

**DOI:** 10.1186/s13550-024-01122-5

**Published:** 2024-07-08

**Authors:** Seweryn Krajewski, Lukasz Steczek, Karina Gotowicz, Urszula Karczmarczyk, Joanna Towpik, Ewa Witkowska-Patena, Krzysztof Łyczko, Maciej Mazur, Przemysław Kozanecki, Joanna Włostowska, Juhani Knuuti, Mirosław Dziuk, Piotr Garnuszek, Cezary Kozanecki

**Affiliations:** 1 Research & Development Centre, Synektik SA, Warsaw, Poland; 2 Department of Chemistry, University of Warsaw, Warsaw, Poland; 3 Radioisotope Centre POLATOM, National Centre for Nuclear Research, Otwock, Poland; 4 Nuclear Medicine Department, Military Institute of Medicine - National Research Institute, Warsaw, Poland; 5 Affidea Poland, Warsaw, Poland; 6 Institute of Nuclear Chemistry and Technology, Warsaw, Poland; 7 Turku PET Centre, Turku University Hospital and University of Turku, Turku, Finland; 8 Department of Clinical Physiology, Nuclear Medicine, and PET, Turku University Hospital, Turku, Finland

**Keywords:** Cardiotracer, Dosimetry, Fluorine-18, PET MPI, Preclinical studies

## Abstract

**Background:**

Positron emission tomography (PET) is now an established diagnostic method for myocardial perfusion imaging (MPI) in coronary artery disease, which is the main cause of death globally. The available tracers show several limitations, therefore, the ^18^F-labelled tracer is in high demand nowadays. The preclinical studies on normal Wistar rats aimed to characterise two potential, novel radiotracers, [^18^F]SYN1 and [^18^F]SYN2, to evaluate which is a better candidate for PET MPI cardiotracer.

**Results:**

The dynamic microPET images showed rapid myocardial uptake for both tracers. However, the uptake was higher and also stable for [^18^F]SYN2, with an average standardized uptake value of 3.8. The biodistribution studies confirmed that [^18^F]SYN2 uptake in the cardiac muscle was high and stable (3.02%ID/g at 15 min and 2.79%ID/g at 6 h) compared to [^18^F]SYN1 (1.84%ID/g at 15 min and 0.32%ID/g at 6 h). The critical organs determined in dosimetry studies were the small intestine and the kidneys. The estimated effective dose for humans was 0.00714 mSv/MBq for [^18^F]SYN1 and 0.0109 mSv/MBq for [^18^F]SYN2. The tested dose level of 2 mg/kg was considered to be the No Observed Adverse Effect Level (NOAEL) for both candidates. The better results were achieved for [^18^F]SYN2, therefore, further preclinical studies were conducted only for this tracer. Radioligand binding assays showed significant responses in 3 from 68 assays: muscarinic acetylcholine M_1_ and M_2_ receptors and potassium channel hERG. The compound was mostly metabolised via an oxidative N-dealkylation, while the fluor substituent was not separated from the molecule.

**Conclusion:**

[^18^F]SYN2 showed a favourable pharmacodynamic and pharmacokinetic profile, which enabled a clear visualization of the heart in microPET. The compound was well-tolerated in studies in normal rats with moderate radiation exposure. The results encourage further exploration of [^18^F]SYN2 in clinical studies.

**Supplementary Information:**

The online version contains supplementary material available at 10.1186/s13550-024-01122-5.

## Background

Ischemic heart disease is a common clinical problem and accurate diagnostic tools are warranted. Among others, nuclear cardiac imaging techniques such as single photon emission computed tomography (SPECT) and positron emission tomography (PET) have been used to detect myocardial ischemia and guiding therapy options such as revascularization [1–4].

The current limiting factor of PET myocardial perfusion imaging (MPI) is availability of tracers, especially in clinical sites without cyclotron [5, 6]. Based on technical superiority of PET over SPECT for myocardial perfusion imaging and feasibility for quantitation of myocardial blood flow in absolute terms [7, 8], the development of new radiotracers for PET MPI may lead to wider use of PET diagnostic imaging in cardiology.

[^82^Rb]Rubidium chloride ([^82^Rb]RbCl) is currently the most commonly used tracer for PET MPI, especially in the USA, but is also registered in some countries of the European Union (Luxembourg, Denmark) [9]. The use of [^82^Rb]RbCl requires a specific generator, which is costly, especially in sites with small number of patients per day. The other PET MPI tracers, [^13^N]ammonia ([^13^N]NH_3_) and [^15^O]H_2_O require on site cyclotron for their production.

The studies on novel PET MPI tracers mainly focus on ^18^F-labelled molecules. ^18^F seems to be an optimal radioisotope for labelling - its chemistry has been well-studied, it can be easily produced in cyclotrons and transported over considerable distances due to its relatively long half-life.

At present, there are several ^18^F-tracers under development, but none of them is currently commercially available [10–12]. One of them, [^18^F]flurpiridaz, completed a phase 3 clinical trials showing greater diagnostic accuracy with PET as compared to SPECT MPI [13]. The comparison of available MPI tracers is shown in Table 1.

**Table 1:** Comparison of available SPECT and PET tracers for myocardial perfusion

	Compound name	Effective dose [14–16] [mSv/MBq]	Recommended dose [17, 18] [MBq]	Sensitivity [13, 19] [%]	Specificity [13, 19] [%]
SPECT	[^201^Tl]Thallium chloride	0.14	37–111	85	80
	[^99m^Tc]Tc-sestamibi	0.0079	150–1200	84	76
	[^99m^Tc]Tc-tetrofosmin	0.0069	150–1200	76	80
PET	[^82^Rb]Rubidium chloride	0.0011	740–1480	87	84
	[^13^N]Ammonia	0.002	400	83	91
	[^15^O]H_2_O	0.0011	400	84	81
	[^18^F]Flurpiridaz	0.015	90–350	80	64

Myocardial uptake of a tracer depends both on its transport into the cell and its subsequent extraction/retention in the cell. The first process depends on the blood flow and the latter on cell membrane integrity and viability. The ideal myocardial perfusion agent should have high first-pass extraction fraction and retention by the myocytes, rapid clearance from blood, low extracardiac uptake, minimal myocardial redistribution, intrinsic chemical stability, and relatively simple radiochemical synthesis and purification process [20]. In addition, the extraction fraction should not be substantially influenced by level of flow as this characteristic makes quantitation of myocardial perfusion more feasible.

There is a group of compounds, widely tested on animals, that show these features − ^18^F-labelled derivatives of triphenylphosphonium cation with a symmetrical structure [10]. Other compounds, which were promising in MPI in clinical studies, are ^18^F-labelled dyes such as rhodamine B and boron-dipyrromethene (BODIPY) derivatives [12, 21]. Taking into account the collected data and that [^13^N]ammonia is an excellent PET MPI tracer, we concluded that desired active pharmaceutical ingredient (API) structure should consist of a nitrogen cationic centre, at least three aromatic rings and be structurally similar to well-known dyes used in staining cell material and organelles. The quaternary ammonium salts, whose main core consists of three fused aromatic rings, commonly used in molecular biology, are ethidium bromide (EthBr) and N-nonyl acridine orange (NOA). We have chosen these compounds as model molecules for developing a novel PET MPI tracer. The EthBr is used to stain the nucleic acids by intercalation between adjacent base pairs. The NOA specifically incorporates into the mitochondrial membrane through primary binding to cardiolipin, a phospholipid present only in the inner layer of the cell membrane [22].

We developed and investigated [^18^F]SYN1 and [^18^F]SYN2, which are ^18^F-labelled derivatives of EthBr and NOA, respectively.

## Methods

The presented preclinical studies were conducted using radioactive tracers [^18^F]SYN1 and [^18^F]SYN2, and also their non-radioactive reference standards (when a high concentration of the compound was needed to carry out the tests).

### Precursor synthesis

**Fig. 1:**
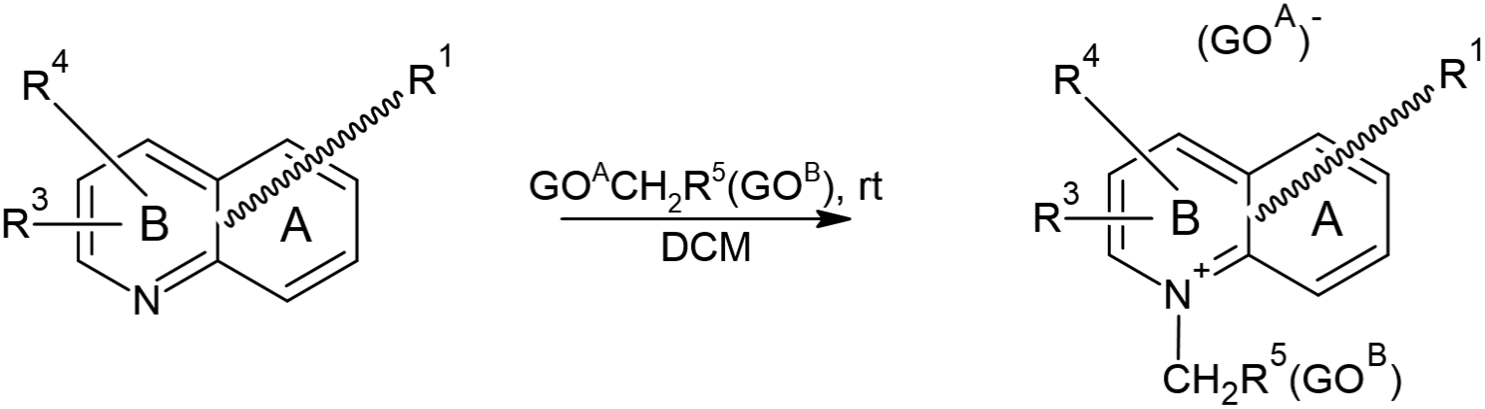
Precursors synthesis scheme

The precursors of [^18^F]SYN1 and [^18^F]SYN2 were synthesized by the method illustrated in Fig. 1. The reagent bearing two groups suitable in a nucleophilic substitution reaction was dissolved in dichloromethane (DCM) and cooled. The phenanthridine derivative or acridine derivative was also dissolved in DCM and added dropwise to the reagent. The contents of the reaction flask were stirred for a given time, and at the end of the reaction, cold Et_2_O was added. The precipitate formed was filtered off under reduced pressure. The details are given in patent EP 3 814 325 B1 [23].

### Radiosynthesis

**Fig. 2:**
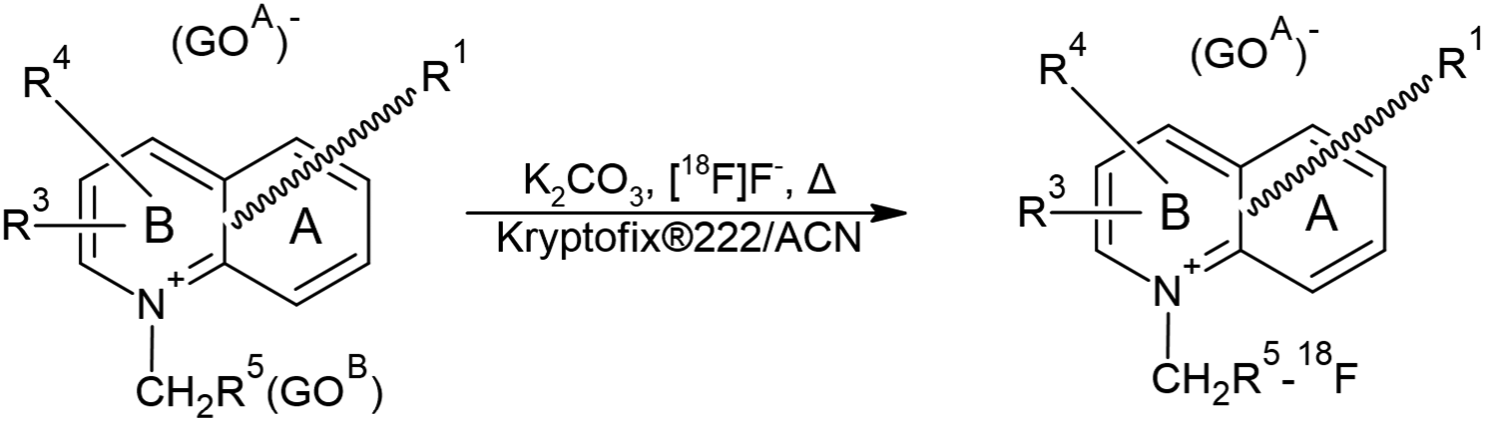
Radiosynthesis of [^18^F]SYN1 and [^18^F]SYN2

The radiosynthesis of [^18^F]SYN1 and [^18^F]SYN2 is based on S_N_2 substitution reaction of [^18^F]F^-^ with the precursor described above (Fig. 2). The process was carried out using a radiosynthesis module (Modular-Lab Standard, Eckert&Ziegler, Germany). The [^18^F]F^-^ was produced using the Eclipse HP cyclotron (11 MeV, Siemens, USA). The usual irradiation conditions were a proton current of 60 µA. The produced [^18^F]F^-^ was captured on the Waters, QMA light SPE column and eluted by a solution of phase-transfer catalyst (Kryptofix^®^ 2.2.2., K_2_CO_3_, water, acetonitrile) into the reactor. Afterwards, the water/acetonitrile mixture was removed under reduced pressure, and azeotropic distillation was carried out. The precursor solution was added to the dried complex of [^18^F]F^-^ with phase-transfer catalyst ([(Kryptofix^®^ 2.2.2.)K]^+^), and the mixture was heated. The crude product was diluted with water/ethanol and purified on a semipreparative HPLC column, where the mobile phase was acetate buffer with the addition of ethanol. The product solution was formulated by dilution with saline to a suitable radioactive concentration and then sterilized by filtration.

### Quality control

The final product was analysed by HPLC to confirm identity and determine radiochemical purity: Xterra, C18 250 × 4.6 mm column (Waters) was used with a flow rate 1 mL/min, gradient elution (phase A – water + 0.05% TFA, phase B – acetonitrile), UV detection at 270 and 500 nm, and on-line radiometric detection; sample volume: 20 µL. Analysis time was ca. 30 min. The free [^18^F]fluoride amount was determined by TLC using silica gel on aluminium plates and 95% ACN/0.9% saline (V:V; 1:1) as mobile phase. GC method was used to determine the ethanol and residual solvents amount using HP-Fast Residual Solvent column (30 m x 0.53 mm, 1.00 μm, Agilent).

The formulation stability was tested for 8 h.

### Animals

All animal procedures were conducted according to the National Legislation and the Council Directive of the European Communities on the Protection of Animals Used for Experimental and Other Scientific Purposes (2010/63/UE) and the “ARRIVE guidelines for reporting animal research” [24].

Wistar rats (6–7 weeks old) were purchased from the M. Mossakowski Institute of Experimental and Clinical Medicine, Polish Academy of Sciences in Warsaw (Poland) (microPET and biodistribution studies), Experimental Medicine Centre at the Medical University in Białystok, Poland (toxicity study) or Envigo RMS Spain S.L. (pharmacokinetics study). On arrival, rats were quarantined and observed for at least five days in groups of not more than five in standard cages in the animal facility of the site conducting study. They were housed in a quiet room under constant conditions (22 ± 2 °C, 55 ± 10% HR, 12 h light/dark cycles) with free access to standard food and water. Veterinarian staff and investigators observed the rats daily to ensure animal welfare and determine if humane endpoints were reached (e.g. hunched and ruffled appearance, apathy, ulceration, severe weight loss).

For the preclinical studies, the animals were randomized into groups containing from 3 to 10 rats per group and time point, depending on the experiment.

### MicroPET

The utility of [^18^F]SYN1 and [^18^F]SYN2 as heart-targeted tracers was tested in male rats using microPET/CT. The scans were performed on a trimodal small animal scanner Albira Si PET/SPECT/CT Preclinical Imaging System (Bruker, Billerica, MA, USA) in a group of Wistar rats anesthetized with isoflurane (induction 4%, maintenance 1.5-2%). Dynamic PET acquisition started at the time of cardiotracer injection via the tail vein ([^18^F]SYN1 11.7 ± 0.5 MBq and [^18^F]SYN2 9.6 ± 0.2 MBq) and lasted for 45 min with time frames of 10 × 30 s, 5 × 60 s, 5 × 120 s and 5 × 300 s.

### Biodistribution and pharmacokinetics

The physiological distribution and pharmacokinetics for [^18^F]SYN1 and [^18^F]SYN2 were examined on rats according to approved protocol. Approximately 0.2 mL/31.1 MBq of compound was administered i.v. At established time points after injection (15 min, 30 min, 1 h, 2 h, 4 h and 6 h), the animals were euthanized by cervical dislocation and dissected. Selected organs and tissues were weighed, and their radioactivity was measured using a NaI(Tl) crystal gamma counter. The results were adjusted for the radioactive decay of ^18^F. The physiological distribution was calculated and expressed in terms of the percentage of administrated dose found in each of the selected organs (%ID) or per gram of tissues/organ (%ID/g) with suitable standards of the injected dose.

The pharmacokinetic study was also conducted using non-radioactive reference standard of [^18^F]SYN2 after a single i.v. administration at a dose of 2 mg/kg in rats. Blood samples of about 0.5 ml were collected in K_3_-EDTA tubes at pre-dose time and 4 min, 15 min, 30 min, 60 min, 4 h, and 24 h after injection (three rats were used at each time point, each rat was bled twice). Blood samples were then centrifuged at approx. 3270 g at 5 ± 3 °C for 10 min to extract plasma, divided in two aliquots and analysed (54 plasma samples in total). The concentrations of SYN2 were determined according to a LC-MS/MS validated method according to the Guidance for Industry (FDA) and Guidance on Validation of Bioanalytical Methods (EMA).

### Dosimetry

The pharmacokinetics data from ex vivo biodistribution studies in rats were extrapolated to humans using the simplest allometric scaling model to estimate an effective dose in mSv/MBq. The dose calculation was done using computer software for internal dose assessment OLINDA/EXM^®^ (Version 1.1, copyright Vanderbilt University, 2007).

### Toxicity

The potential health hazards resulting from exposure to intravenous administration of [^18^F]SYN1 and [^18^F]SYN2 were investigated. Extended single dose toxicity study after administration of 2 mg/kg b.w. of either test item (non-radioactive reference standards, SYN1 or SYN2) in a solution of 0.9% NaCl or only 0.9% NaCl into 30 males and 30 females of Wistar rats was investigated. The rats were euthanized 24 h (*n* = 40) and 14 days (*n* = 20) post-administration. Throughout the experiment, the animals’ behaviour was observed and their weight recorded. The overview of animal groups used in the study is presented in Table 2.

**Table 2:** Animal groups overview in the toxicity studies

Group		Number of animals		Administered item	Animal euthanasia
		males	females		
Control	0–1	10	10	0.9% NaCl solution	24 h post-administration
	0–2	5	5		14 days post-administration
Treated	1	10	10	Test item solution in 0.9% NaCl at dose 2 mg/kg b.w.	24 h post-administration
	2	5	5		14 days post-administration

### Pharmacodynamics

The possibility of pharmacological interactions of SYN2 was assessed in vitro in radioligand binding assays on key pharmacological target classes including GPCRs, drug transporters, ion channels, nuclear receptors and enzymes (LeadProfilingScreen SafetyScreen, PP68, Panel of 68 receptors, Eurofins Panlabs, Inc.) [25]. There was only one SYN2 concentration of 1 µM used in the study. The significant response, which confirmed selective binding with target, was defined as ≥ 50% inhibition or stimulation.

### Metabolites

The metabolite profiling and characterization experiment of SYN2 molecule (in vitro MetID, Admescope) was conducted to evaluate potential metabolic pathways. In order to characterize the metabolites of SYN2, the compound was incubated in cryopreserved rat, dog and human hepatocytes. Prior to the incubation, the hepatocytes were thawed, suspended in Williams medium E and then centrifuged to receive a target density of about 1 million cells/ml. A SYN2 solution of 1 µM was then added to the hepatocyte suspension and incubated at 37 °C for 0, 30 and 60 min. The incubations were stopped by the addition of acetonitrile, then frozen and centrifuged to remove precipitated proteins. Supernatants were then analysed with ultra-performance liquid chromatography (UPLC) and high-resolution mass spectrometry (HRMS).

### Statistical analysis

The results of radiopharmaceutical uptake are presented as a percentage of the dose administered per gram of tissue (%ID/g) in the form of an average with standard deviation (%ID/g; mean ± standard deviation (SD)). Where applicable, statistical analysis was performed with one-way analysis of variance (ANOVA) using GraphPad Prism software (GraphPad Software, La Jolla, CA, USA).

The results of toxicity studies conducted for SYN1 and SYN2 were analysed using Excel 2013 and STATISTICA 10.0.PL. The normality distribution was tested with Shapiro-Wilk test. For homogeneity of variance Brown-Forsythe test has been used. For normal distribution and homogeneous variances results, Student’s t-test was used. In the absence of normal distribution, Mann-Whitney test was used. In the case of non-homogenous variance, Cochrane-Cox test was used.

IC_50_ values from pharmacodynamics assessment were determined by a non-linear, least squares regression analysis using MathIQ^™^ (ID Business Solutions Ltd., UK). Where inhibition constants (K_i_) are presented, the K_i_ values were calculated using the equation of Cheng and Prusoff [26] using the observed IC_50_ of the tested compound, the concentration of radioligand employed in the assay, and the historical values for the K_D_ of the ligand (obtained experimentally at Eurofins Panlabs, Inc.). Where presented, the Hill coefficient (n_H_), defining the slope of the competitive binding curve, was calculated using MathIQ^™^.

Compound Discoverer (CD) was used for data mining with manual confirmation for metabolite profiling and characterization.

All of the obtained results were considered statistically significant for *p* < 0.05.

## Results

The sterile cardiotracers solutions with radiochemical purity > 95% were achieved. The ethanol content was not higher that 10% and residual solvents concentration was within the *Ph.Eur.* limits. The stability of the formulation within 8 h was confirmed.

### MicroPET

**Fig. 3:**
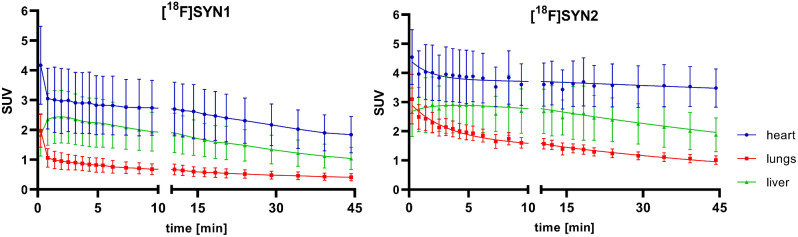
MicroPET time-activity curves (TACs) of heart, lungs and liver for [^18^F]SYN1 and [^18^F]SYN2. Results are presented as mean standardized uptake value ± standard error of measurement

**Fig. 4:**
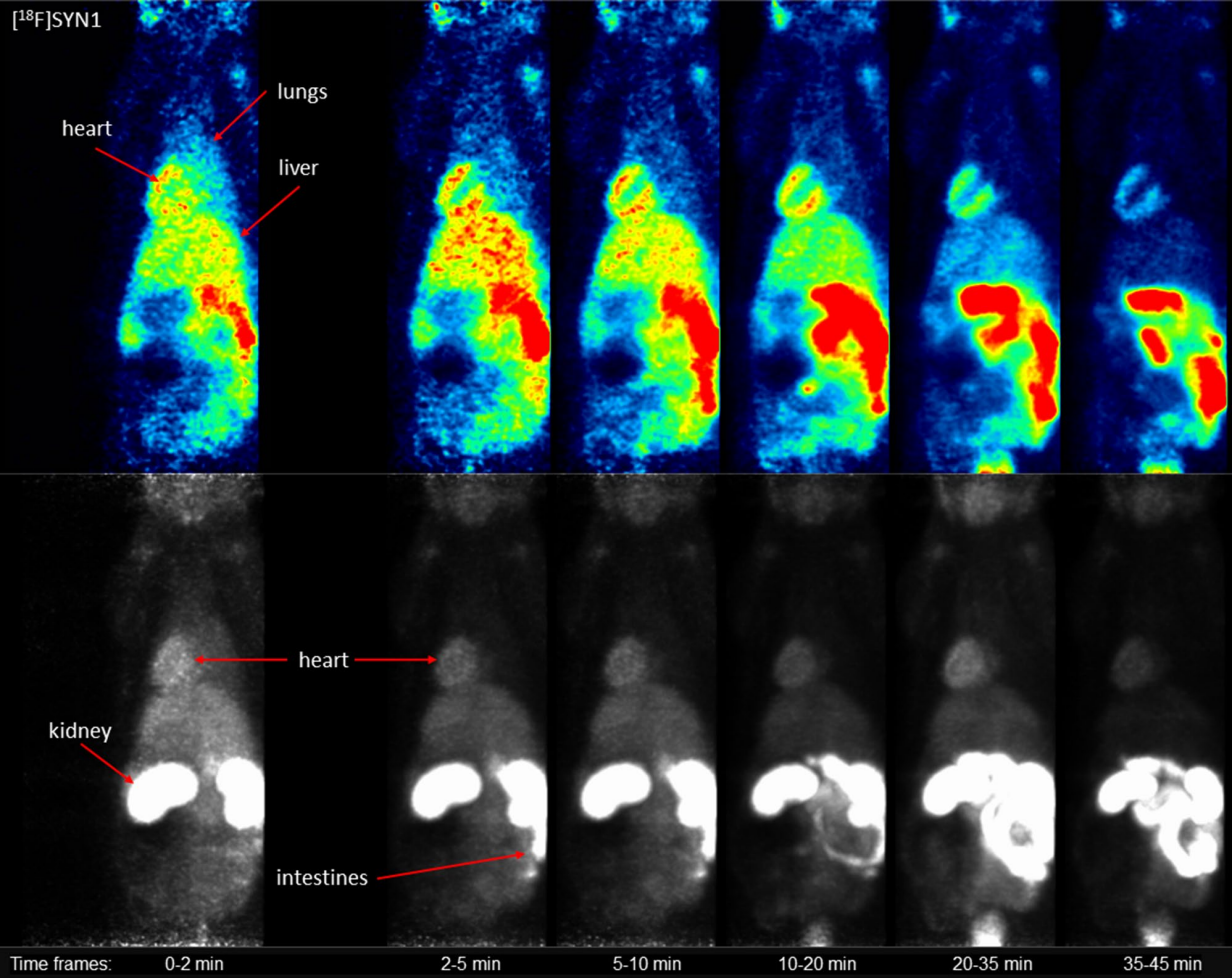
Representative microPET images of [^18^F]SYN1: slice (top) in the coronal plane and maximum intensity projection (bottom). Images represent the summation of time frames

**Fig. 5:**
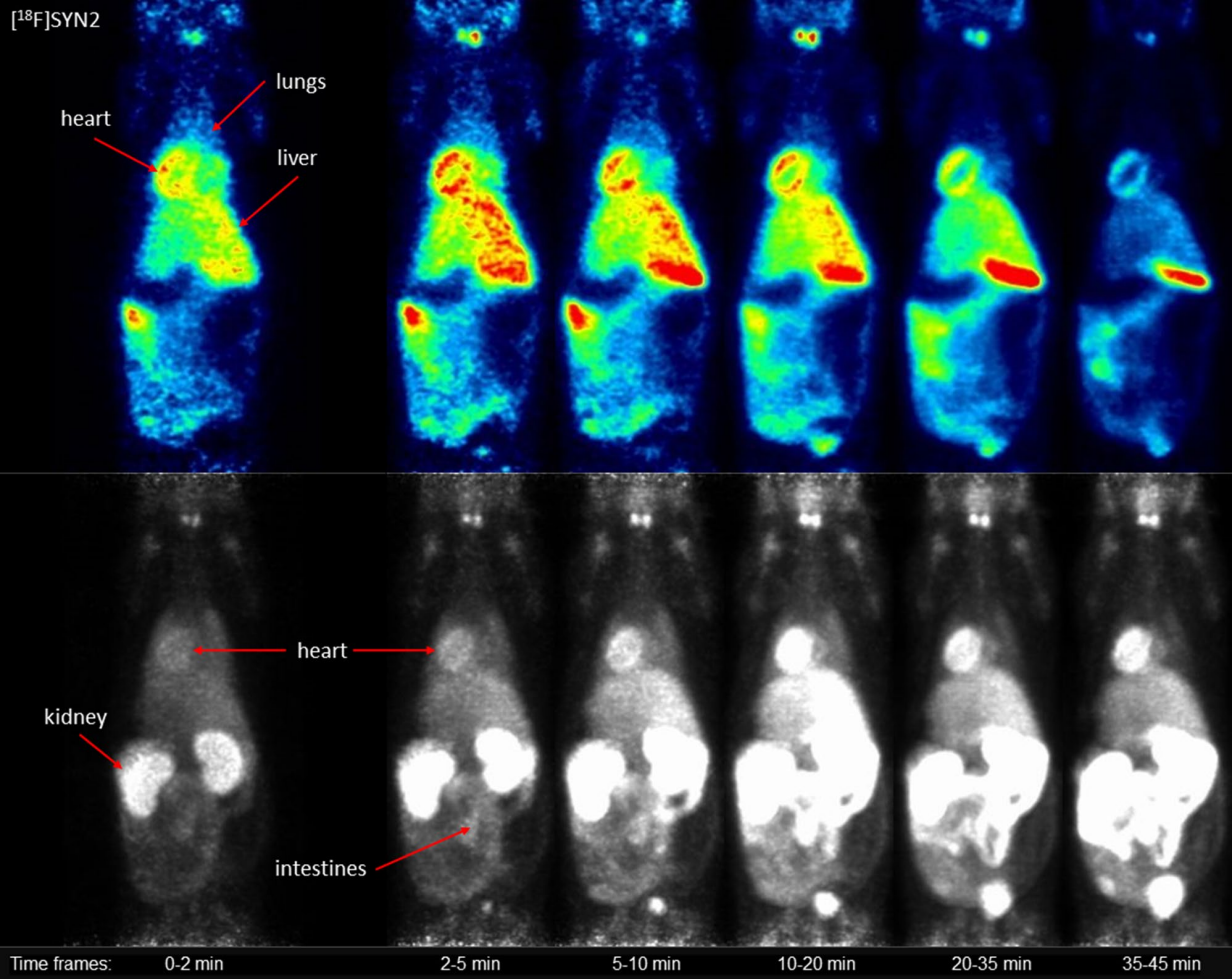
Representative microPET images of [^18^F]SYN2: slice (top) in the coronal plane and maximum intensity projection (bottom). Images represent the summation of time frames

The dynamic microPET data (Fig. 3) showed higher accumulation of [^18^F]SYN2 compared to [^18^F]SYN1 in the organs of interest: heart, liver and lungs. The uptake of [^18^F]SYN2 in the myocardium was stable and high, the SUV value was 4.0 at the beginning and slightly decreased in time reaching plateau at a value of 3.5 after 6 min. In comparison, [^18^F]SYN1 uptake was lower (SUV 3.0 at the beginning) and decreased in time (SUV 1.8 after 45 min). The accumulation in the liver was higher for [^18^F]SYN2 with slower elimination compared to [^18^F]SYN1, the SUV_max_ of 2.9 was reached around 4 min with decrease to 1.9 at 45 min for [^18^F]SYN2, whilst for [^18^F]SYN2 the SUV_max_ was 2.4 at 2 min decreased to 1.0, respectively). However, the distribution ratio between heart and liver was comparable for both radiotracers and was equal to 1.2–1.3 for the first 10 min. The radioactivity uptake and retention in the lungs were also higher for [^18^F]SYN2. Small intestine and bladder were clearly visible 5 min post-injection (Figs. 4 and 5) and the SUV values increased in these organs with time.

### Biodistribution and Pharmacokinetics

[^18^F]SYN1 and [^18^F]SYN2 generally showed a similar distribution. The critical organ for [^18^F]SYN1 and [^18^F]SYN2 was the intestine, with higher radioactivity uptake for [^18^F]SYN1. The accumulation in the intestine increased over 15 min to 6 h from 22.0 ± 6.1 to 52.3 ± 8.4%ID for [^18^F]SYN1 and from 17.1 ± 1.8 to 33.4 ± 1.4%ID for [^18^F]SYN2. High and significant difference in compound accumulation was noted in the kidneys, for which [^18^F]SYN2 accumulated at twice the rate of [^18^F]SYN1 compound. For example, at 15 min the accumulation was 13.4 ± 4.0 and 5.4 ± 1.5%ID for [^18^F]SYN2 and [^18^F]SYN1, respectively, and at 6 h the value was 3.4 ± 0.4 and 0.5 ± 0.1%ID, respectively. All other organs examined retained only small amounts of test items, at level below 1% ID/g for [^18^F]SYN1 and 2%ID/g for [^18^F]SYN2. Full data of tissue distribution are listed in Supplemental Tables 1–4.

**Fig. 6:**
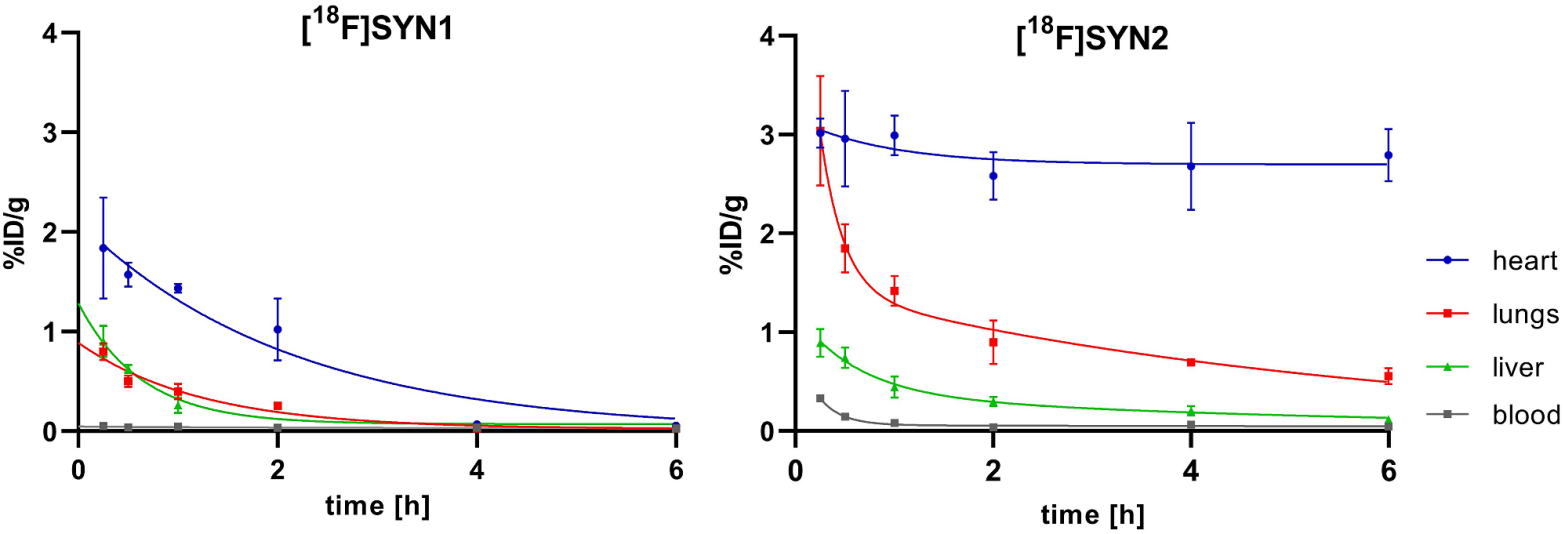
Uptake of [^18^F]SYN1 and [^18^F]SYN2 in selected organs, ex vivo biodistribution study

The uptake of [^18^F]SYN2 in the myocardium was stable and higher than 2.5%ID/g for up to 6 h, compared to [^18^F]SYN1 which was between 2 and 1%ID/g up to 2 h, and then it dramatically decreased below 0.4%ID/g. Figure 6 shows comparative value uptake (%ID/g) in the heart as a target organ and in the lungs and the liver as critical organs during imaging.

**Fig. 7:**
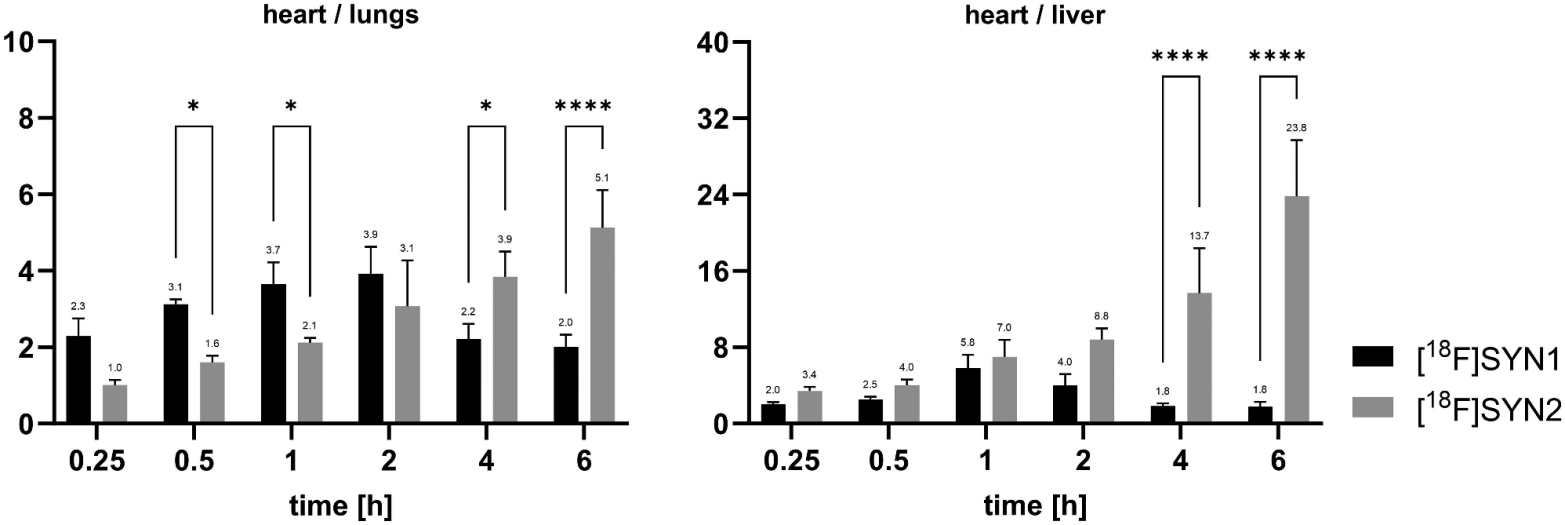
Comparison of heart/lungs and heart/liver ratios between [^18^F]SYN1 and [^18^F]SYN2, calculation based on %ID/g

Furthermore, we found that for [^18^F]SYN2, the ratio of radioactivity accumulation in the heart to the liver and lungs was more favourable from an imaging point of view (Fig. 7). The most favourable and statistically significant ratios were observed after 2 h of radiotracer administration.

**Fig. 8:**
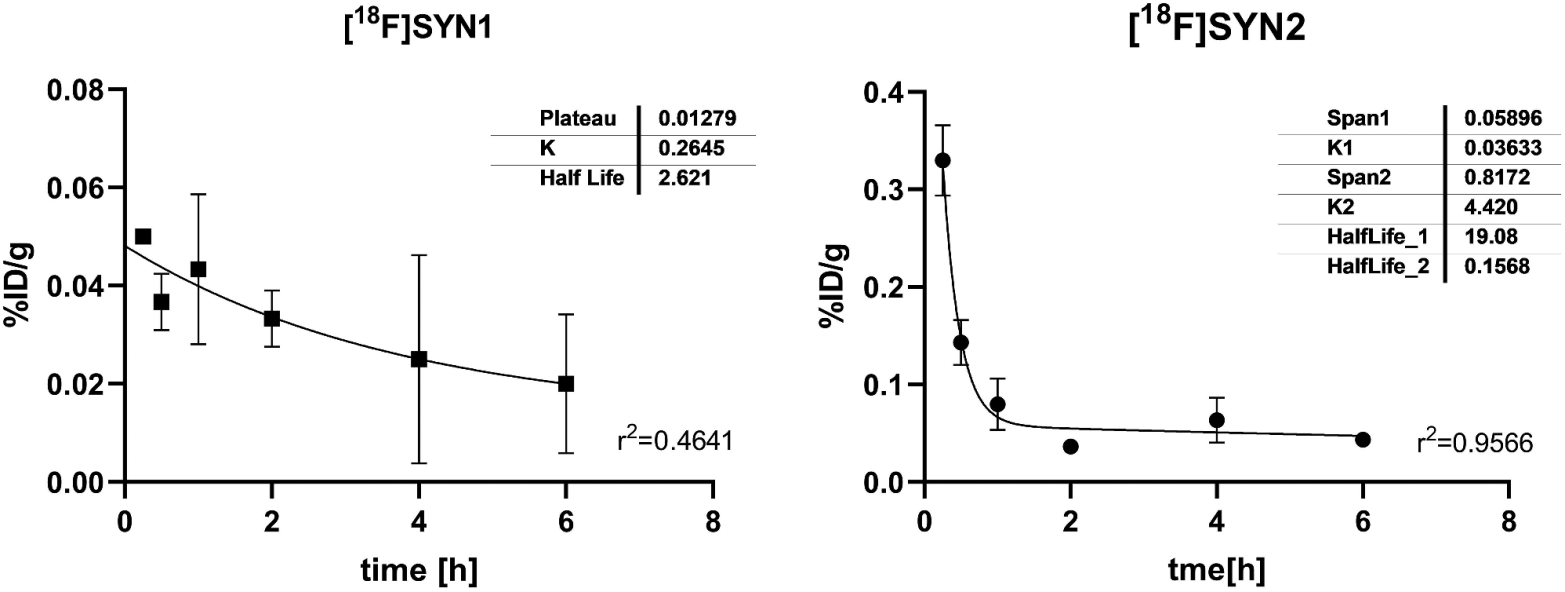
Blood activity clearance. The fit of a one-phase and two-phase exponential model

The blood pharmacokinetics of [^18^F]SYN1 and [^18^F]SYN2 as a one-phase and two-phase exponential decay model, respectively, are shown in Fig. 8. For [^18^F]SYN2 the initial fast decrease mainly represents the equilibrium between the intravascular and extravascular components, and the second slower phase represents the metabolism.

It is worth mentioning that half-life parameters for [^18^F]SYN1 and [^18^F]SYN2 calculated from blood activity clearance differ from those presented in Table 3 which is most likely related to the administered dose. The additional pharmacokinetic (PK) parameters for both radiolabelled compounds are presented in the Table 4.

**Fig. 9:**
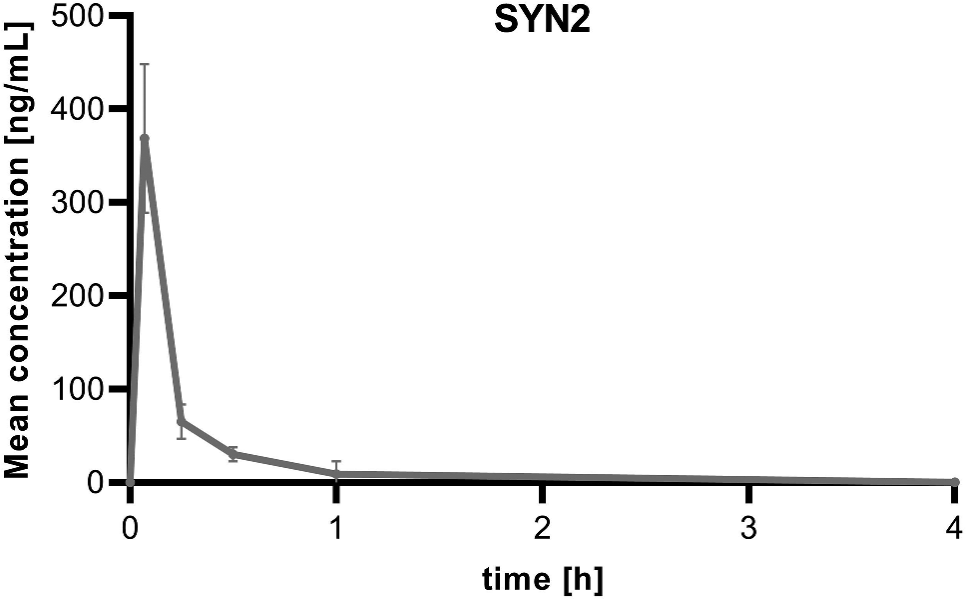
Plasma concentration time profile for the unlabelled reference standard of [^18^F]SYN2

The pharmacokinetics of [^18^F]SYN2 was also measured using its non-radioactive reference standard SYN2. The tested item was quantifiable in plasma from the first sampling time point (4 min) down to 4 h. The plasma levels showed a rapid decay up to 1 h post injection. The elimination half-life was 0.26 h. The estimated plasma clearance was 2.59 L/h/kg. High total plasma clearance and low volume of distribution were obtained in the rats (Table 3; Fig. 9).

**Table 3:** Pharmacokinetic parameters of SYN2 in the rat

T_max_ (h)	C_max_ (ng/mL)	T_last_ (h)	C_last_ (ng/mL)	AUC_t_ (h·ng/mL)	V_z_ (L/kg)	Cl (L/h/kg)	T_½λz_ (h)
0.07	368.67	1.00	8.95	73.75	0.99	2.59	0.26

T_max_ – time to peak plasma concentration, C_max_ – peak plasma concentration, T_last_ – time to last quantifiable plasma concentration, C_last_ – last quantifiable plasma concentration, AUC_t_ – area under the plasma concentration-time curve from time 0 to the time of the C_last_, calculated by the linear trapezoidal linear/log rule, V_z_ – volume of distribution at the terminal phase, Cl – plasma clearance, T_1/2λz_ – terminal half-life

**Table 4:** Pharmacokinetic parameters for [^18^F]SYN1 and [^18^F]SYN2 in the rat

	T_max_ [h]	C_max_ [ng/mL]	AUC_t_ [h·ng/mL]	V_z_ [L/kg]	Cl [L/h/kg]	T_½_ [h]^*^
[^18^F]SYN1	0	40.99	73	3.12	0.037	57.8
[^18^F]SYN2	0	76.39	421	1.43	0.012	79.9

T_1/2_ –half-life calculated from Eq. (0.693 · V_Z_ · AUC)/dose

**Table 5:** Toxicity study results

Conducted tests	SYN1*	SYN2*
*Clinical examinations*		
Mortality	-	-
Clinical observations	-	-
Body weight	-	-
*Clinical-chemical examinations*		
Haematological investigations	-	-
Biochemical investigations	+	+
Enzymatic investigations	+	+
*Post mortem examination*		
Gross examination	+	+
Weight of internal organs	-	+
Histopathological examinations	+	+

* “+” – statistically significant changes were observed between control and treated group

“-” – no statistically significant changes were observed

### Safety

#### Toxicity

The objective of the toxicity study was to obtain information on health hazards resulting from a single exposure due to the intravenous administration of each of test item at dose of 2 mg/kg b.w., which is 1000-fold higher than the predicted maximum dose in humans.

The effects of each of test item on animals’ body weight, clinical parameters as well as haematological, biochemical, enzymatic parameters, gross and histopathological lesions in tissues and internal organs were tested. Summary of collected data regarding the toxicity study of SYN1 and SYN2 is presented in Table 5 comparing if there were statistically significant changes observed between control and treated group.

#### Dosimetry

This work estimated the human absorbed radiation dose based on a detailed description of activity organ distribution data for normal rats. After administration of [^18^F]SYN1 and [^18^F]SYN2, the animals were placed in holding cages which guaranteed their freedom of movement. Therefore, Table 6 compares estimated absorbed doses (mSv/MBq) in human organs for [^18^F]SYN1 and [^18^F]SYN2. Additionally, the obtained theoretical values for examined cardiotracers were compared with [^18^F]flurpiridaz absorbed dose calculated in exercise stress subjects in humans (Table 6) [14].

**Table 6:** Estimates of absorbed doses (mSv/MBq) for [^18^F]SYN1 and [^18^F]SYN2 in human organs after extrapolation of biodistribution data in rats and absorbed doses for [^18^F]flurpiridaz derived from human studies [14]

Organs	Absorbed dose per administrated activity [mSv/MBq]		
	[^18^F]SYN1	[^18^F]SYN2	[^18^F]Flurpiridaz
Adrenals	7.54E-03	8.06E-03	1.40E-02
Brain	2.00E-03	2.84E-03	1.10E-02
Breasts	5.17E-03	2.93E-03	1.00E-02
Gallbladder Wall	8.21E-03	8.01E-03	1.50E-02
Lower Large Intestine Wall	7.99E-03	1.18E-02	1.40E-02
Small Intestine	1.96E-02	1.84E-02	1.40E-02
Stomach Wall	7.88E-03	1.19E-02	2.40E-02
Upper Large Intestine Wall	9.18E-03	2.01E-02	1.40E-02
Heart Wall	1.39E-02	4.15E-02	3.90E-02
Kidneys	1.85E-02	7.04E-02	2.70E-02
Liver	7.63E-03	1.17E-02	1.50E-02
Lungs	6.95E-03	2.16E-02	1.20E-02
Muscle	6.17E-03	1.03E-02	1.20E-02
Ovaries	8.64E-03	7.10E-03	1.40E-02
Pancreas	1.11E-02	2.85E-02	1.50E-02
Red Marrow	6.08E-03	4.77E-03	1.50E-02
Osteogenic Cells	9.42E-03	4.02E-03	2.00E-02
Skin	4.79E-03	2.65E-03	9.00E-03
Spleen	6.09E-03	2.13E-02	1.30E-02
Testes	5.99E-03	4.97E-03	1.10E-02
Thymus	6.50E-03	5.48E-03	1.30E-02
Thyroid	6.18E-03	2.49E-02	1.40E-02
Urinary Bladder Wall	7.37E-03	5.29E-03	1.60E-02
Uterus	8.55E-03	6.27E-03	1.40E-02
Total Body	6.49E-03	7.67E-03	1.20E-02
Effective Dose Equivalent [mSv/MBq]	9.31E-03	1.71E-02	1.60E-02
Effective Dose [mSv/MBq]	7.14E-03	1.09E-02	1.50E-02

The data in Table 6 shows that [^18^F]SYN1 compound resulted in 34% less effective dose compared to [^18^F]SYN2 and 52% as compared to [^18^F]flurpiridaz. However, the dose absorbed in the heart wall was also clearly lower for [^18^F]SYN1 (0.0139, 0.0415 and 0.0390 for [^18^F]SYN1, [^18^F]SYN2 and [^18^F]flurpiridaz, respectively), indicating a disadvantage of the [^18^F]SYN1 compound.

The highest radiation-absorbed doses per unit-administered activity (mSv/MBq) of [^18^F]SYN2 were calculated for kidneys (0.0704), heart wall (0.0415) and pancreas (0.0285), in contrast to [^18^F]flurpiridaz for which the highest values were for heart wall (0.039), kidneys (0.027) and stomach wall (0.024). It’s worth mentioning that the high value of the absorbed dose for [^18^F]SYN2 in kidneys should not influence imaging quality of heart, which is related to the position of organs in the human body. However, obtained absorber dose for [^18^F]SYN2 in kidneys is twice that of [^99m^Tc]Tc-MIBI (0.036) [15], but at that level does not induce kidney toxicity.

The obtained preclinical data suggest that [^18^F]SYN2 is a well-tolerated PET radiopharmaceutical with favourable radiation dosimetry profile and that is suitable for clinical cardiac imaging.

### Pharmacodynamics

Radioligand binding assays showed significant responses (≥ 50% inhibition or stimulation) in three out of 68 assays: muscarinic acetylcholine M_1_ receptor (58% inhibition), muscarinic M_2_ acetylcholine receptor (68% inhibition) and potassium channel hERG (57% inhibition). The range of responses for the remaining 65 assays was from − 12 to 43 (mean: 7.63, median: 4, IQR [-1] − 13). Functional assays at several concentrations of SYN2 for IC_50_ or EC_50_ determination performed subsequently for muscarinic M_2_ and hERG showed that SYN2 has an antagonistic effect on both targets with an IC_50_ about 1 µM.

### Metabolites

**Fig. 10:**
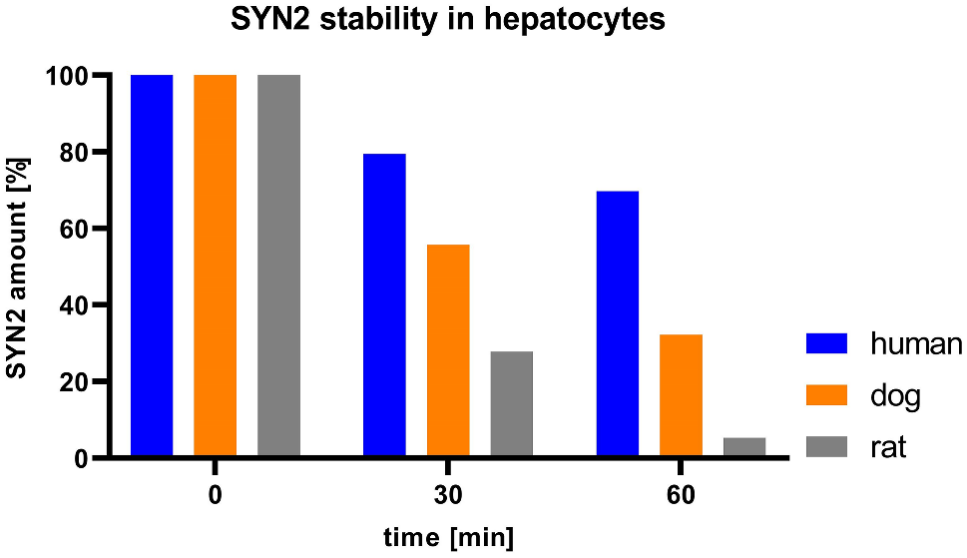
Stability of SYN2 in human, dog and rat hepatocytes

Twelve metabolites were identified in the study by analysis of high-resolution LC-MS/MS data and characterized in vitro in cryopreserved rat, dog and human hepatocytes (Supplemental Table 5). The products of reactions such as N-dealkylation, oxidation, acetylation and glucuronidation were identified and the fluorine atom was generally not detached. The metabolism of SYN2 in human hepatocytes was rather low in comparison to rat and dog (32% and 5% of the parent, respectively), the unchanged SYN2 was predominant after 60 min and was equal to 70% (Fig. 10). The major metabolite in dog cells was metabolite M1 and in rat cells metabolite M2. These metabolites appeared to be major metabolites in human hepatocytes with relative abundance of 19% and 8%, respectively at 60 min. In dog hepatocytes, the N-dealkylated metabolites M1, M2 and M3 were predominant at 60 min, respectively 58%, 53%, 46% of relative abundance. In rat cells the major metabolite was M2 (almost 7 times that of the parent at 60 min). The structures of M4, M5, M6, M7 and M8 were not fully elucidated due to very low contents, and generally may represent products of dealkylated/N-acetylated/oxidated forms of SYN2.

## Discussion

We assessed biodistribution, pharmacokinetics, elimination and safety of [^18^F]SYN1 and [^18^F]SYN2, ^18^F-labelled phenanthridine and acridine derivatives, respectively, candidate radiotracers for PET MPI. The further preclinical studies, pharmacodynamics and metabolism, were conducted only for [^18^F]SYN2.

Myocardium was clearly visible in microPET for both radiotracers, however time-activity curves showed stable heart radioactivity uptake only for [^18^F]SYN2 with high SUV values of 4.0-3.5 throughout the scan time (about 45 min). The microPET images suggested that both cardiotracers were excreted via the biliary and urinary pathway. MicroPET also showed negligible skeletal activity, indicating that ^18^F is not hydrolysed or released in the form of [^18^F]fluoride ion in vivo for both compounds (Figs. 4 and 5).

The biodistribution studies demonstrated very similar results to microPET findings. They confirmed the higher than [^18^F]SYN1 and sustained myocardial uptake of [^18^F]SYN2 with values of about 3.0%ID/g within 1-hour post-injection and then 2.6–2.8%ID/g from 2 to 6 h. The uptake in the liver was also higher for [^18^F]SYN2, and similarly to microPET results, the heart/liver ratio was also higher. The distribution profiles confirmed that both radiotracers were excreted via the biliary and urinary pathway (Supplemental Tables 1–4).

The biodistribution results of [^18^F]SYN2 can be compared with other tracers for myocardial perfusion, like [^18^F]flurpiridaz (former name ^18^F-BMS-747158-02) and [^99m^Tc]Tc-sestamibi. For example, at 1 h post-injection the accumulation in cardiac tissue was comparable with [^18^F]flurpiridaz, 3.0 ± 0.2 vs. 3.3 ± 0.3%ID/g, respectively, and higher than [^99m^Tc]Tc-sestamibi (2.0 ± 0.3%ID/g). However, the heart/liver ratio was the highest for [^18^F]SYN2, 7.0 ± 1.8 vs. 3.7 ± 0.2 and 2.4 ± 0.2, respectively. The heart/lungs ratio was on the other hand the lowest for [^18^F]SYN2, ratio of 2.1 ± 0.2 vs. 12.7 ± 1.4 and 5.9 ± 0.5, respectively [27]. Furthermore, the heart/blood ratio was significantly higher for [^18^F]SYN2 in comparison with [^18^F]flurpiridaz (37 vs. 13) [28]. The observed differences between the biodistribution parameters of ^18^F-labelled cardiotracer, [^18^F]SYN2 and [^18^F]flurpiridaz, are not significant enough to draw conclusion, which tracer is the best for myocardial perfusion imaging, however both has advantage over [^99m^Tc]Tc-sestamibi. Therefore, further clinical studies are warranted.

The approximated estimation of [^18^F]SYN2 dosimetry in humans showed that the organ receiving the highest estimated absorbed dose were the kidneys at 0.070 mSv/MBq, followed by the heart wall at 0.042 mSv/MBq (Table 6). The value for heart wall is comparable to [^18^F]flurpiridaz, the new cardiac perfusion agent now in phase III clinical trials (0.027 mSv/MBq and 0.039 mSv/MBq, respectively) [14]. The estimation for [^18^F]SYN1, the second tested compound, showed that the small intestine received the highest estimated absorbed dose at 0.020 mSv/MBq, followed by the kidney at 0.019 mSv/MBq and heart wall with much lower value compared to [^18^F]SYN2 and [^18^F]flurpiridaz at 0.014 mSv/MBq (Table 6). The effective dose of [^18^F]SYN1 was the lowest, followed by [^18^F]SYN2 and then [^18^F]flurpiridaz – 0.0072 mSv/MBq vs. 0.0109 mSv/MBq vs. 0.0150 mSv/MBq, respectively [14]. For comparison, effective doses for ^82^Rb and [^13^N]ammonia, short lived cardiotracer, are much lower, 0.0011 mSv/MBq and 0.002 mSv/MBq, respectively [15, 16]. The in-house data of estimated dosimetry of the ^18^F-labelled compounds presented in this paper are derived from allometrically scaled results from rat studies to humans. This is an important limitation and may result in discrepancies from the dosimetry determined in direct human studies. Such studies are planned.

SYN1 and SYN2 were well-tolerated and only slight abnormalities were reported in the toxicological study. The minor biochemical abnormalities were within the normal limits for Wistar rats [29]. Minimally increased incidence of basophilic tubules in the kidneys of male rats (without other renal changes) might have been the result of spontaneously occurring chronic progressive nephropathy, one of the most common spontaneous diseases in rats correlated with age [30]. Hepatocyte karyomegaly and foci of single hepatocyte necrosis are a normal incidental finding in the rats and may occur spontaneously with age [31]. In conclusion, the histopathological changes found in all study groups were background findings and although they might have been related to SYN1 or SYN2 administration, the dose level of 2 mg/kg b.w. is considered to be “no observed adverse effect level” (NOAEL).

The high and stable uptake of the [^18^F]SYN2 in the heart muscle together with good heart/liver distribution ratio and low toxicity make this radiotracer suitable for the heart muscle imaging. Therefore, further preclinical studies were conducted only with the [^18^F]SYN2.

The possible pharmacological interactions of SYN2 were tested and significant inhibition was observed for 3 out of 68 targets at a concentration of 1 µM – muscarinic acetylcholine receptors M_1_ and M_2_ as well as hERG. The determined values indicate that SYN2 show high affinity to hERG receptors and moderate to muscarinic receptors [32]. The muscarinic acetylcholine receptors are multifunctional, they are responsible for regulating heart rate, smooth muscle contraction, glandular secretion and many fundamental functions of the central nervous system [33], while hERG plays an important role in electrical activity in the heart [34]. The affinity of acridine derivatives to hERG receptors was already confirmed in the range of 0.2–18 µM [35]. The structure that has positively charged nitrogen atom and hydrophobic aromatic ring system can be deducted as a potential hERG blocker using QSAR strategy. The results of analysing different database highlighted that hERG inhibitors tend to have a larger molecular weight, higher hydrophobicity, more cations and less basic substituents [35]. Similar conclusion can be applied to muscarinic receptors, e.g. the tacrine, acridine derivative, is the known antagonist [36].

Despite the wide abundance of mentioned targets in the human body (including heart) any interactions of SYN2 are considered unlike, as it reached low plasma concentrations [37–40]. We demonstrated in normal Wistar rats that SYN2 reached C_max_ of 0.19 µM after i.v., injection of 2 mg/kg with a half-life of 0.26 h. After injection of a proposed maximum limit dose of 100 µg (2 µg/kg for a 50-kg patient), we expect C_max_ of 1.1 nM after body surface area correction, which is unreliable to lead to any measurable biological effects.

The metabolic profiles in human, dog and rat hepatocytes were compared to evaluate potential metabolism pathways. Firstly, the drug product usually undergoes reaction of functionalization, very frequent to be more polar. The metabolites found in dog, rat and human hepatocytes suggest that SYN2 is mainly oxidatively N-dealkylated in a subsequent mode forming M1 and M2 metabolites. In addition, in dog and rat hepatocytes the M3 metabolite was observed as well. Only the structures of M1, M2 and M3 were confirmed by MS fragmentation spectra to high content. However, it seems that hydroxylation can occur as well as metabolites M5, M6 were detected [41], though the forming of N-oxide cannot be excluded. Secondly, metabolites are usually coupled with an endogenous molecule undergoing acetylation or (N, O)-glucuronidation reaction. Metabolites M7, M8 are N-acetylation products similarly to proflavine [42]. The product of the last reaction is only observed in rat and dog hepatocytes presumably to slower metabolism in human cells. The gathered data indicated that main metabolism of SYN2 proceeded via oxidative N-dealkylation(s) followed by acetylation yielded polar metabolites that can be excreted in urine and/or bile. The other pathway seen in dog and rat hepatocytes via hydroxylation and O-glucuronidation appear to be of lower significance. Similarly, the dealkylation at N-acridine atom, followed by cleavage of fluorine atom is of lower significance due to the fact that quaternary amines are more stable than tertiary or secondary amines toward N-dealkylation.

## Conclusions

[^18^F]SYN2 showed favourable pharmacodynamic and pharmacokinetic profile in normal Wistar rats. The tracer, with its rapid plasma clearance, high and stable uptake in the myocardium and favourable heart to lung ratio, allowed for a clear visualization of the heart in microPET. [^18^F]SYN2 was safe, well-tolerated and showed moderate radiation exposure comparable with other MPI PET tracers. Promising results of preclinical evaluation of [^18^F]SYN2 encourage its further exploration in clinical studies.

### Electronic supplementary material

Below is the link to the electronic supplementary material.

Supplementary Material 1

## Data Availability

The datasets used and/or analyzed during the current study are available from the corresponding author on reasonable request.

## Abbreviations

[^18^F]SYN1: ^18^F-labelled derivative of ethidium bromide
[^18^F]SYN2: ^18^F-labelled derivative of acridine orange
%ID: Percentage of injected dose per organ
%ID/g: Percentage of injected dose per gram of tissue
API: Active pharmaceutical ingredient
AUCt: Area under the plasma concentration curve from time 0 to the time of the C_last_ calculated by the linear trapezoidal linear/log rule
BODIPY: Boron-dipyrromethene
CAD: Coronary artery disease
Cl: Plasma clearance
C_last_: last quantifiable plasma concentration
C_max_: Peak plasma concentration
CPN: Chronic Progressive Nephropathy
DCM: Dichloromethane
EMA: European Medicines Agency
EthBr: Ethidium bromide
FDA: Food and Drug Administration
HRMS: High-resolution mass spectrometry
IC_50_: Half maximal inhibitory concentration
ICA: Invasive Coronary Angiography
IHD: Ischemic heart disease
MBF: Myocardial blood flow quantification
MPI: Myocardial perfusion imaging
NOA: N-nonyl acridine orange
NOAEL: No observed adverse effect level
PCM: Progressive Cardiomyopathy
PET: Positron emission tomography
RCP: Radiochemical purity
SD: Standard deviation
SPECT: Single photon emission computed tomography
SYN1: Non-radioactive reference standard of [^18^F]SYN1
SYN2: Non-radioactive reference standard of [^18^F]SYN2
T_1/2λz_: Terminal half-life
T_last_: Time to last quantifiable plasma concentration
T_max_: Time to peak plasma concentration
UPLC: Ultra-performance liquid chromatography
WHO: World Health Organization
V_z_: Volume of distribution at the terminal phase
