# Supplementary material for: Preclinical evaluation of [18F]SYN1 and [18F]SYN2, novel radiotracers for PET myocardial perfusion imaging
Source: EJNMMI Res. 2024 Jul 8;14:63. doi: 10.1186/s13550-024-01122-5 (PMC11231114; doi:10.1186/s13550-024-01122-5)
Supplement: Supplementary file 1 — Supplementary Material 1 [file 13550_2024_1122_MOESM1_ESM.pdf]

Supplementary information

Preclinical evaluation of [^{18}F]SYN1 and [^{18}F]SYN2, novel radiotracers for PET myocardial perfusion imaging

Seweryn Krajewski[1,*], Lukasz Steczek[1], Karina Gotowicz[1,2], Urszula Karczmarczyk[3], Joanna Towpik[1], Ewa Witkowska-Patena[4,5], Krzysztof Łyczko[6], Maciej Mazur[2], Przemysław Kozanecki[1], Joanna Włostowska[1], Juhani Knuuti[7,8], Mirosław Dziuk[4,5], Piotr Garnuszek[3], Cezary Kozanecki[1]

[1]Research & Development Centre, Synektik SA, Warsaw, POLAND

[2]Department of Chemistry, University of Warsaw, Warsaw, POLAND

[3]Radioisotope Centre POLATOM, National Centre for Nuclear Research, Otwock, POLAND

[4]Nuclear Medicine Department, Military Institute of Medicine - National Research Institute, Warsaw, POLAND

[5]Affidea Poland, Warsaw, POLAND

[6]Institute of Nuclear Chemistry and Technology, Warsaw, POLAND

[7]Turku PET Centre, Turku University Hospital and University of Turku, Turku, FINLAND

[8]Department of Clinical Physiology, Nuclear Medicine, and PET, Turku University Hospital, Turku, FINLAND

Table 1. Results of the [^{18}F]SYN1 biodistribution in Wistar rats (%ID, mean ± SD)

	**Mean %ID (±SD) versus time after dosing**					
**Organ**	15 min	30 min	1 h	2 h	4 h	6 h
Brain	0.12 (0.02)	0.10 (0.01)	0.11 (0.04)	0.12 (0.02)	0.10 (0.02)	0.13 (0.04)
Intestine	21.99 (6.12)	37.52 (3.47)	36.53 (0.09)	38.09 (6.08)	49.69 (2.22)	52.29 (8.36)
Stomach	0.64 (0.14)	0.56 (0.01)	0.51 (0.22)	0.36 (0.07)	0.26 (0.08)	0.22 (0.04)
Heart	1.15 (0.30)	0.97 (0.16)	0.93 (0.09)	0.54 (0.09)	0.28 (0.02)	0.20 (0.04)
Kidneys	5.41 (1.54)	2.56 (0.13)	1.72 (0.21)	0.89 (0.15)	0.61 (0.14)	0.46 (0.11)
Liver	5.83 (0.73)	4.68 (0.37)	1.82 (0.38)	1.44 (0.14)	1.27 (0.19)	1.07 (0.12)
Lungs	0.91 (0.08)	0.58 (0.04)	0.48 (0.07)	0.29 (0.04)	0.22 (0.04)	0.18 (0.01)
Pancreas	0.75 (0.12)	0.83 (0.17)	0.57 (0.03)	0.54 (0.09)	0.35 (0.02)	0.26 (0.04)
Spleen	0.22 (0.05)	0.22 (0.03)	0.12 (0.00)	0.10 (0.03)	0.11 (0.04)	0.08 (0.01)
Urine	2.59 (3.67)	12.32 (2.84)	11.40 (5.06)	14.79 (1.81)	18.27 (1.22)	18.52 (6.70)
Rest of the body	48.19 (6.74)	44.44 (2.07)	43.93 (3.98)	48.19 (6.74)	44.44 (2.08)	43.93 (3.97)

Table 2. Results of the [^{18}F]SYN2 biodistribution in Wistar rats (%ID, mean ± SD)

	**Mean %ID (±SD) versus time after dosing**					
**Organ**	15 min	30 min	1 h	2 h	4 h	6 h
Brain	0.23 (0.09)	0.19 (0.01)	0.22 (0.12)	0.12 (0.01)	0.25 (0.04)	0.21 (0.02)
Intestine	17.12 (1.81)	18.27 (1.40)	20.18 (3.19)	25.93 (2.92)	30.51 (0.47)	33.42 (1.37)
Stomach	1.99 (0.68)	1.54 (0.12)	1.84 (0.47)	1.66 (0.16)	1.94 (0.38)	1.53 (0.15)
Heart	2.15 (0.18)	2.19 (0.26)	2.23 (0.11)	2.03 (0.23)	2.15 (0.08)	2.27 (0.21)
Kidneys	13.35 (3.97)	14.01 (3.78)	12.91 (2.36)	9.65 (1.42)	5.20 (0.36)	3.44 (0.37)
Liver	7.57 (0.96)	6.87 (1.10)	3.66 (0.83)	2.46 (0.28)	1.71 (0.27)	1.07 (0.11)
Lungs	3.88 (0.36)	2.64 (0.47)	1.89 (0.23)	1.25 (0.31)	0.89 (0.02)	0.78 (0.14)
Pancreas	1.33 (0.23)	0.97 (0.32)	0.93 (0.01)	0.94 (0.22)	0.84 (0.35)	0.80 (0.05)
Spleen	1.01 (0.20)	0.79 (0.16)	0.60 (0.10)	0.44 (0.04)	0.32 (0.03)	0.29 (0.02)
Urine	0.46 (0.31)	0.53 (0.25)	1.59 (0.30)	3.94 (3.35)	12.42 (1.40)	15.27 (0.87)
Rest of the body	51.01 (2.66)	51.34 (1.73)	46.30 (1.89)	44.09 (5.00)	44.50 (2.38)	44.67 (1.68)

Table 3. Results of biodistribution of the [^{18}F]SYN1 in Wistar rats (%ID/g, mean ± SD)

	**Mean %ID/g (±SD) versus time after dosing**					
**Organ**	**15 min**	**30 min**	**1 h**	**2 h**	**4 h**	**6 h**
Blood	0.05 (0.00)	0.04 (0.00)	0.04 (0.02)	0.03 (0.01)	0.03 (0.00)	0.03 (0.00)
Brain	0.07 (0.01)	0.06 (0.01)	0.06 (0.02)	0.07 (0.02)	0.06 (0.01)	0.08 (0.02)
Intestine	1.51 (0.54)	2.02 (0.20)	2.09 (0.37)	2.81 (0.13)	3.10 (0.27)	3.26 (0.59)
Stomach	0.49 (0.14)	0.43 (0.05)	0.38 (0.14)	0.29 (0.06)	0.20 (0.06)	0.17 (0.04)
Heart	1.84 (0.51)	1.57 (0.12)	1.43 (0.05)	1.02 (0.31)	0.38 (0.03)	0.32 (0.07)
Kidneys	3.53 (1.30)	1.43 (0.20)	0.98 (0.12)	0.65 (0.10)	0.37 (0.09)	0.31 (0.09)
Liver	0.90 (0.16)	0.63 (0.04)	0.26 (0.08)	0.25 (0.01)	0.19 (0.02)	0.16 (0.01)
Lungs	0.80 (0.09)	0.50 (0.06)	0.40 (0.08)	0.26 (0.03)	0.18 (0.01)	0.15 (0.03)
Pancreas	0.97 (0.12)	0.91 (0.15)	0.91 (0.01)	0.77 (0.10)	0.55 (0.03)	0.41 (0.11)
Spleen	0.37 (0.02)	0.25 (0.04)	0.20 (0.03)	0.19 (0.06)	0.18 (0.04)	0.11 (0.04)

Table 4. Results of biodistribution of the [^{18}F]SYN2 in Wistar rats (%ID/g, mean ± SD)

	**Mean %ID/g (±SD) versus time after dosing**					
**Organ**	15 min	30 min	1 h	2 h	4 h	6 h
Blood	0.33 (0.04)	0.14 (0.02)	0.08 (0.02)	0.04 (0.00)	0.06 (0.02)	0.04 (0.01)
Brain	0.13 (0.15)	0.11 (0.00)	0.12 (0.06)	0.07 (0.00)	0.13 (0.02)	0.13 (0.01)
Intestine	0.98 (0.10)	1.29 (0.40)	1.04 (0.18)	1.50 (0.24)	1.88 (0.10)	2.00 (0.49)
Stomach	1.65 (0.94)	1.11 (0.08)	1.33 (0.33)	1.23 (0.22)	1.58 (0.25)	1.12 (0.12)
Heart	3.02 (0.15)	2.96 (0.49)	2.99 (0.20)	2.58 (0.24)	2.68 (0.44)	2.79 (0.27)
Kidneys	7.07 (1.77)	7.22 (2.09)	6.76 (1.23)	5.10 (0.85)	2.86 (0.08)	1.69 (0.22)
Liver	0.89 (0.14)	0.74 (0.11)	0.45 (0.11)	0.30 (0.04)	0.21 (0.05)	0.12 (0.02)
Lungs	3.04 (0.55)	1.85 (0.24)	1.42 (0.15)	0.90 (0.22)	0.70 (0.02)	0.55 (0.08)
Muscle	0.82 (0.13)	0.46 (0.11)	0.58 (0.18)	0.41 (0.09)	0.51 (0.04)	0.53 (0.05)
Pancreas	2.22 (0.24)	1.75 (0.56)	1.63 (0.16)	1.55 (0.36)	1.56 (0.06)	1.27 (0.10)
Spleen	1.96 (0.51)	1.59 (0.41)	1.15 (0.23)	0.84 (0.11)	0.66 (0.06)	0.56 (0.02)

Table 5. Summary of potential metabolites of SYN2 in hepatocytes from human (H), dog (D) and rat (R) together with relative abundance of metabolites calculated as % of parent (SYN2)

**Metabolite**	**R_t (min)**	**Accurate m/z difference between parent and metabolite**	**Proposed bio-transformation**	**Matrix detected (H/D/R)**	**Relative abundance of metabolites calculated as % of parent (SYN2)**					
					**Human**		**Dog**		**Rat**	
					30 min	60 min	30 min	60 min	30 min	60min
**SYN2 (Parent)**	5.25	Parent	Parent structure	H/D/R	100	100	100	100	100	100
**M1**	4.99	-14.0154	Demethylation	H/D/R	10	19	34	58	94	137
**M2**	4.70	-28.0311	Two demethylations	H/D/R	4	8	22	53	115	732
**M3**	4.36	-42.0467	Three demethylations	H/D/R	1	3	9	46	27	666
**M4**	5.00	-46.0216	Dealkylation	H/D/R	1	1	Trace	Nd	Trace	Nd
**M5**	4.11	-12.036	Two demethylations, and an oxidation	H/D/R	1	1	2	7	7	80
**M6a**	3.84	-26.0518	Three demethylations, and an oxidation	H/D/R	Trace	Trace	Trace	4	Trace	23
**M6b**	4.24	-26.0519		H/D/R	Trace	Trace	Trace	7	Trace	Trace
**M7a**	4.10	-0.0363	Three demethylations, and acetylation; or one demethylation, and oxidation	H/D/R	2	3	Trace	4	Trace	10
**M7b**	4.23	-0.0361		H/D/R	0	1	Trace	2	Trace	15
**M8a**	3.87	-14.0518	Four demethylations, and acetylation; or two demethylations, and oxidation	H/D/R	1	1	Trace	1	Nd	8
**M8b**	4.00	-14.0518		H/D/R	1	1	3	8	3	52
**M9**	4.13	192.0271	Oxidation and glucuronidation	D/R	Nd	Nd	1	6	Nd	1

Nd – not detected
